# Managing zoonotic infectious diseases in Africa: The key role approach

**DOI:** 10.4102/ojvr.v91i2.2195

**Published:** 2024-11-07

**Authors:** Abdalla A. Latif

**Affiliations:** 1 School of Life Sciences, University of KwaZulu-Natal, Durban, South Africa

One Health is an integrated, unifying approach that aims to sustainably balance and optimise the health of humans, animals (domestic and wildlife), and ecosystems. They are closely linked and interdependent. The One Health approach mobilises multiple sectors, disciplines, and communities at varying levels of society to work together at community, national, regional, and global levels. Thus, multiple sectors are required to work together to address health challenges. As outlined in the One Health Joint Plan of Action, the One Health approach collectively addresses emerging and re-emerging diseases, neglected and tropical diseases, antimicrobial resistance, food security, and environmental challenges.

Across the African continent, there are multiple effective national, regional, and sub-regional networks that are centred around One Health initiatives. These networks work to mobilise policymakers and One Health professionals to improve disease surveillance, outbreak preparedness and response, and information sharing. While many of these groups are aware of each other’s efforts and priorities, there is significant opportunity for better coordination and integration to share best practices, lessons learned, and resources to promote the next generation of Pan-African One Health leadership. With the mission to produce a cross-border collaborative and information-sharing network for One Health Initiatives in Africa, the African One Health Network (AfOHNet) aims to identify and connect One Health stakeholders, building strategic networks, partnerships, and fostering education on One Health issues to support a paradigm shift in information sharing, active health interventions, collaborations, and demonstration projects. Importantly, AfOHNet aims to mainstream One Health into a practical initiative whose implementation can be appreciated by all stakeholders.

The AfOHNet Inaugural Workshop serves as a Pan-African forum for networking and multidisciplinary engagement, with the mission of strengthening disease detection, diagnoses, and reporting in African nations collaborating across the continent. The Inaugural AfOHNet Workshop, held in Accra, Ghana, in 2022 with the theme ‘Managing zoonotic infectious diseases in Africa: The key role approach’ was aimed to provide AfOHNet members a Pan-African networking opportunity for multidisciplinary engagement with a mission of strengthening disease detection, diagnoses, and reporting in African nations collaborating across the continent. The workshop included a mixture of keynote panel presentations, dedicated thematic sessions, and lively discussions and debates on the challenges that impact building an integrated, non-duplicative One Health scientific network by sharing best practices and lessons learned from established One Health initiatives. The 5-day conference also included breakout workshops, and working group panels to enrich regional collaborations, networking, and encourage and promote a new generation of young One Health leadership across Africa. The primary workshop topics addressed, included making the connection between One Health science and global health security, coronavirus disease 2019 (COVID-19) resiliency and recovery success stories, innovative One Health approaches to bio-surveillance, the best practices of One Health risk communication, activation of a One Health Network: moving from theory to practice and new technologies for low-resource settings.

The published proceedings in this Special Issue outlined and discussed various issues related to One Health. A National Veterinary Research Institute that had experience in viral diagnosis responded to the public health challenge for the diagnosis of COVID-19; over 33 000 samples were processed within 6 months. This work was possible through the collective utilisation of their human and material resources, including biosafety facility, equipment, diagnostic reagents, and consumables provided by international partners and collaborators. Thereafter, many field and laboratory projects were jointly implemented between veterinary and collaborating sectors, which brought together professionals in the health, education and socio-sciences.

The COVID-19 pandemic has provided the opportunity for One Health to move from theory to practice. The question of how to accelerate the scaling up of the One Health approach through the synergy between policies, researchers, providers and communities is clarified in West African countries in one publication. Examples of specific diseases in relation to One Health were the objective in three publications: Leptospira spp. in the wildlife, livestock and humans’ interface, rabies vaccination status of dogs in households and antibiotic resistance and mitigation through One Health.

The author wishes to acknowledge Dr Misheck Mulumba, Chair of the Steering Committee, and members of the steering committee of the African One Health Network (AfOHNet) workshop ‘Inaugrural AfOHNet Workshop’, held in Accra, Ghana, 03 October 2022 to 07 October 2022.

